# Perspectives on the adaptation of Japanese plum-type cultivars to reduced winter chilling in two regions of Spain

**DOI:** 10.3389/fpls.2024.1343593

**Published:** 2024-04-17

**Authors:** Brenda I. Guerrero, Erica Fadón, M. Engracia Guerra, Javier Rodrigo

**Affiliations:** ^1^ Departamento de Ciencia Vegetal, Centro de Investigación y Tecnología Agroalimentaria de Aragón (CITA), Zaragoza, Spain; ^2^ Facultad de Ciencias Agrotecnológicas, Universidad Autónoma de Chihuahua, Chihuahua, Mexico; ^3^ Instituto Agroalimentario de Aragón-IA2 (CITA-Universidad de Zaragoza), Zaragoza, Spain; ^4^ Área de Fruticultura Mediterránea, Instituto de Investigaciones Agrarias “Finca La Orden-Valdesequera”, Centro de Investigaciones Científicas y Tecnológicas de Extremadura (CICYTEX) A-V, Badajoz, Spain

**Keywords:** chilling requirements, heating requirements, *Prunus salicina*, dormancy, dynamic model, flower buds, climate projections

## Abstract

Japanese plum, like other temperate fruit tree species, has cultivar-specific temperature requirements during dormancy for proper flowering. Knowing the temperature requirements of this species is of increasing interest due to the great genetic variability that exists among the available Japanese plum-type cultivars, since most of them are interspecific hybrids. The reduction of winter chilling caused by climate change is threatening their cultivation in many regions. In this work, the adaptation perspectives of 21 Japanese plum-type cultivars were analyzed in two of the main plum-growing regions in Spain, Badajoz and Zaragoza, to future climate conditions. Endodormancy release for subsequent estimation of chilling and heat requirements was determined through empirical experiments conducted during dormancy at least over two years. Chill requirements were calculated using three models [chilling hours (CH), chilling units (CU) and chilling portions (CP)] and heat requirements using growing degree hours (GDH). Chilling requirements ranged 277-851 CH, 412-1,030 CU and 26-51 CP, and heat requirements ranged from 4,343 to 9,525 GDH. The potential adaption of the cultivars to future warmer conditions in both regions was assessed using climate projections under two Representative Concentration Pathways (RCP), RCP4.5 (effective reduction of greenhouse gas emissions) and RCP8.5 (continuous increase in greenhouse gas emissions), in two time horizons, from the middle to the end of 21st century, with temperature projections from 15 Global Climate Models. The probability of satisfying the estimated cultivar-specific chilling requirements in Badajoz was lower than in Zaragoza, because of the lower chill availability predicted. In this region, the cultivars analyzed herein may have limited cultivation because the predicted reduction in winter chill may result in the chilling requirements not being successfully fulfilled.

## 1 Introduction

Japanese plum (hybrids of *Prunus salicina* Lindl.), like other temperate fruit tree species, enters a period of dormancy in late autumn to survive at the low temperatures of winter and blooms at the end of winter in most of the growing areas (Lang et al., 1985; Guerra and Rodrigo, 2015). Dormancy has been defined as the absence of signs of visible growth in any plant structure containing a meristem, and three several phases can been distinguished: (1) endodormancy, regulated by internal physiological factors of the affected plant structure, (2) paradormancy, regulated by physiological factors external to the structure, such as apical dominance, and (3) ecodormancy, regulated by environmental factors (Lang et al., 1987). During endodormancy, meristems become more resistant to adverse conditions caused by low temperatures, but exposure to certain amount of chilling is necessary to overcome the dormant state. Chilling requirements are specific to each cultivar and refer to the duration and depth of chilling required during endodormancy (Jansson and Douglas, 2007). Once trees fulfill their chilling requirements, they enter the ecodormancy phase, which is influenced by external environmental factors (Lang et al., 1985). During this phase, trees must be exposed to a period of warm temperatures for bud break and flowering to occur (Brown and Kotob, 1956; Lang et al., 1987; Guerra and Rodrigo, 2015; Fadón et al., 2020b).

Knowing the agroclimatic requirements for proper flowering, including chilling during endodormancy and heating during ecodormancy, is essential for predicting whether a cultivar can successfully adapt to a cultivation area (Fadón and Rodrigo, 2018). This is of increasing interest due to the reduction of winter chilling caused by climate change in many regions (Luedeling, 2012; Delgado et al., 2021; Fernandez et al., 2022) and the great genetic variability that exists among the available Japanese plum-type cultivars since most of them are interspecific hybrids (Guerra et al., 2009). To estimate both chilling and heating requirements, first it is necessary to determine when the endodormancy is released, and then quantify the previous accumulated chilling. The most common approach is an empirical technique that involves assessing the phenology and growth of flower buds on shoots collected sequentially during winter after a period in a forcing chamber with controlled photoperiod and warm temperatures (Brown and Kotob, 1956; Tabuenca, 1967; Campoy et al., 2012; Ruiz et al., 2018; Fadón et al., 2018a; 2020a). Once the date of endodormancy release is established, it is necessary to quantify the chill accumulated until then to estimate the chilling requirements, and the heat accumulated from endodormancy release until flowering to estimate heat requirements. To determine which temperatures are effective in each phase, different models have been proposed to quantify chill during endodormancy and heat during ecodormancy (Campoy et al., 2011; Fadón & Rodrigo, 2018). The three most used models for quantifying chilling were developed to predict peach phenology and propose different units. The Weinberger model establishes that a chilling hour (CH) corresponds to an hour between 0 and 7.2°C (Weinberger, 1950). The Utah model establishes that one chilling unit (CU) corresponds to an hour between 2.5 and 9.1°C; 0.5 CU corresponds to an hour in two ranges: from 1.5 to 2.4°C and from 9.2 to 12.4°C, while CU accumulation is null at temperatures below 1.4°C and between 12.5-15.9°C, and negative for temperatures equal to or greater than 16°C (Richardson et al., 1974). The dynamic model proposes that chilling accumulation occurs in a two-phase process, in which warm temperatures can nullify the effect of previous cold temperatures, and is expressed in chilling portions (CP) (Fishman et al., 1987). On the other hand, for quantifying warm temperatures, the most used model is Growing Degree Hours (GDH). One GDH is defined as one hour at 1°C above the base temperature of 4.5°C, so they are calculated by subtracting 4.5°C from each hourly temperature between 4.5°C and 25°C. All temperatures above 25°C are considered to have an effect equal to 25°C (Richardson et al., 1974).While numerous studies have been conducted on determining agroclimatic requirements in stone fruit trees (Fadón et al., 2020b and cites therein), available information of Japanese plum is based on only two studies conducted in two areas in Spain, Zaragoza (eight cultivars) (Tabuenca, 1967) and Murcia (11 cultivars) (Ruiz et al., 2018), with only two cultivars, Golden Japan and Santa Rosa, being analyzed in both studies.

In recent years, Japanese plum is undergoing significant varietal renewal, with the release of a number of new cultivars from breeding programs in various countries (Topp et al., 2012; Guerra and Rodrigo, 2015; Guerrero et al., 2020). The introduction of these new releases into commercial orchards is leading to production problems in some cases due to a lack of knowledge regarding their agronomic behavior and adaptability to each cultivation zone (Guerra and Rodrigo, 2015; Guerrero et al., 2020). The cultivars whose agroclimatic requirements have been studied so far represent a very small percentage of those currently available, making it necessary to study other cultivars to determine their adaptability to each cultivation zone, especially in warmer areas with mild winters (Fadón et al., 2020b). Limited winter chill prevents overcoming dormancy, which can lead to delayed flowering, erratic and scarce flowering, or flower sterility, ultimately leading to significant yield losses (Erez, 2000). To predict the effects of global warming on winter chill availability, we use a chilling model alongside climate model projections for the upcoming decades of the 21st century, considering various emissions scenarios or representative concentration pathways (RCPs). The aim is to generate chilling forecasts and align them with the chilling requirements of the cultivars to assess their suitability (Rodríguez et al., 2020). This approach has been used in previous studies on other fruit tree species, which have shown that certain cider apple cultivars and traditional sweet cherry cultivars, with high chilling requirements, will see their production compromised in Asturias (Spain) (Delgado et al., 2021) and Zaragoza (Spain) (Fadón et al., 2023), respectively, according to the climatic projections assayed. Characterizing agroclimatic requirements is crucial in temperate fruit breeding programs to develop new cultivars well adapted to future climatic conditions (Delgado et al., 2021).

In this work, we forecast the adaptation of 21 Japanese plum-type cultivars to future climate conditions in four locations from two of the main plum growing regions of Spain, Badajoz (Guadajira) and Zaragoza (Caspe, La Almunia and Montañana). To this end, we first experimentally characterized the endodormancy and ecodormancy phases, and then calculated the chill requirements using three chilling models (Chilling Hours, Utah, and Dynamic) and the heat requirements using one forcing model (Growing Degree Hours). We assessed the expected winter chill accumulation in the two regions for the middle (2050) and end (2085) of 21st century, considering two global warming scenarios (Representative Concentration Pathways 4.5 and 8.5) using temperature projections from 15 Global Climate Models. Finally, we estimated the probability of chilling fulfillment of each cultivar by comparing their chill requirements to the estimated future chill accumulation in each region.

## 2 Materials and methods

### 2.1 Plant material

Twenty-one commercial Japanese plum-type cultivars were analyzed (**Supplementary Table S1**), sourced from collections located in two of the main plum growing regions of Spain, Badajoz and Zaragoza, with different agroclimatic conditions. In the region of Badajoz, plant material was obtained from 21 cultivars in the collection of CICYTEX-La Orden in Guadajira (38°51’9.6” N, 6°40’15.6” W, and 214 m altitude). In the region of Zaragoza, plant material was obtained from three different locations: eight cultivars from the collection of CITA in Montañana (41°43’37.1” N, 0°47’21.0” W, and 220 m altitude), nine cultivars from the collection of AFRUCCAS in Caspe (Asociación de Fruticultores de la Comarca de Caspe) (41°18’56.5” N, 0°05’06.9” E, and 172 m altitude), and four cultivars from the collection of “Viveros Mariano Soria” in La Almunia (41°28’37.4” N, 1°22’26.8” W, and 366 m altitude). The experiments were conducted during the winters from 2017 to 2021, with at least two years of data collected at each location: Montañana (2017-2018; 2018-2019; 2019-2020; 2020-2021), Caspe (2017-2018; 2018-2019; 2019-2020), La Almunia (2019-2020; 2020-2021), and Guadajira (2019-2020; 2020-2021).

### 2.2 Flowering phenology

The phenology of each cultivar was monitored during flowering in the studied years. The duration of the flowering period was established from the opening of the first flowers to the last. Full bloom was considered when at least 50% of the flowers (F50) were at the F phenological stage (Baggiolini, 1952), corresponding to stage 65 on the BBCH scale (Meier, 2001; Fadón et al., 2015).

### 2.3 Endodormancy release determination

Weekly sampling of shoots was conducted from early November to early February. On each sampling day, 5 shoots (20-30 cm in length and 4-8 mm in diameter with at least 10 floral buds each) from each cultivar were collected. A fresh cut was made underwater at the base of the shoots, and they were placed in floral foam with water and kept for 8 days in a forcing chamber, with a 12-hour light photoperiod provided by daylight lamps (6500 K, Osram L58W/865 and T5 L21W/865) and a temperature of 22 ± 1°C (Fadón et al., 2018b).

The date of endodormancy release was determined based on the growth and phenological development of the buds. On each sampling day and for each cultivar, the phenological stage of all buds on each shoot was recorded, and 10 randomly selected flower buds were weighed at the beginning (day 0) and the end (day 8) of the period in the forcing chamber. The date of endodormancy release was established when the weight of the floral buds increased by at least 30% after 8 days in the forcing chamber (Ruiz et al., 2018; Fadón et al., 2018b).

### 2.4 Chilling requirements estimation

To estimate chilling requirements, semi-hourly temperature data were recorded at stations located in each location: station BA205-La Orden in Guadajira, Badajoz (REDAREX, 2023), and stations 31 (Épila), 34 (Montañana) and 38 (Caspe) in Zaragoza (ORESA, 2023). The chill accumulation from October 1st to the date of endodormancy release in each cultivar was quantified using the three most commonly used models in fruit trees (Fadón et al., 2020b). The Weinberger model defines one Chilling Hour (CH) as an hour between 0 and 7.2°C (Hutchins, 1932, as cited by Weinberger, 1950). The Utah model proposes that one Chilling Unit (CU) corresponds to an hour between 2.5 and 9.1°C, 0.5 CU corresponds to an hour in two ranges: from 1.5 to 2.4°C and from 9.2 to 12.4°C, while CU accumulation is zero at temperatures less than 1.4°C and between 12.5-15.9°C, and negative for temperatures equal to or greater than 16°C (Richardson et al., 1974). The dynamic model considers that chill accumulation occurs in a two-phase process, where warm temperatures can counteract the effect of previous cold temperatures and is expressed in Chilling Portions (CP) (Fishman et al., 1987).

### 2.5 Heating requirements estimation

Heating requirements were determined using the model proposed by Richardson (1974), by summing up Growing Degree Hours (GDH) accumulated from the end of endodormancy to full bloom (F50). One GDH is defined as 1 hour at 1°C above the base temperature of 4.5°C. GDHs were calculated by subtracting 4.5°C from each hourly temperature between 4.5°C and 25°C. All temperatures above 25°C are considered to have the same effect as 25°C, resulting in a maximum accumulation of 20.5 GDH (Richardson et al., 1974).

### 2.6 Estimation of chill availability for historic and future weather scenarios

We assessed the likely performance of the Japanese plum-type cultivars under various scenarios in the regions of Zaragoza and Badajoz. We evaluated observations recorded *in-situ* as well as simulated temperature data generated for historic and future periods. For the region of Zaragoza, historic temperature records were used from the meteorological station placed in Montañana due to present a longer period of temperature records (1973-2023). The historic temperatures for the region of Badajoz were recorded in Talavera La Real (1973-2023). To better identify long-term trend and reduce the effect of year-to-year variation, historic weather scenarios that characterize typical agroclimatic conditions were generated following the methods described by Fernandez et al. (2020) and Delgado et al. (2021) with minor modifications. In brief, we produced representative temperature scenarios for the years 1980, 1990, 2000, 2010, and 2020 using temperature data recorded on site. For each of these years, typical mean daily minimum and maximum temperatures were determined for each month by applying a 15-year running mean function across all recorded monthly extreme temperatures for the respective month for all years on record. By using functions in the chillR package (Luedeling et al., 2021), we then applied these five typical temperature scenarios to the RMAWGEN weather generator (Cordano and Eccel, 2016) to produce 100 replicates of plausible winter seasons for each scenario year.

The same procedure described above was used to predict future agroclimatic conditions in both regions, Zaragoza and Badajoz. As input for the weather generator in this case, we used monthly means of daily temperature extremes obtained from the ClimateWizard database (Girvetz et al., 2009), which is maintained by the International Center for Tropical Agriculture (CIAT). ClimateWizard offers temperature projections by 15 Global Climate Models (GCMs) for two Representative Concentration Pathway scenarios (RCP4.5 and RCP8.5) and for several time horizons. RCP4.5 and RCP8.5 represent total radiative forcing of 4.5 W m^−2^ and 8.5 W m^−2^ caused by atmospheric greenhouse gases by 2100 (IPCC, 2014). While the RCP4.5 scenario illustrates a situation in which authorities institute some policies that are effective to reduce emissions, RCP8.5 is a scenario with very high greenhouse gas emissions (IPCC, 2014). For both RCPs, we obtained future temperature scenarios for the middle (represented by mean conditions between 2035 and 2065) and for the end of the 21st century (mean conditions between 2070 and 2100). These two-time horizons were represented by their central years 2050 and 2085, respectively. We used 60 future scenarios (each representing one combination of GCM, RCP and time horizon) to feed the weather generator and produce 100 replicates of plausible winter seasons given the expected environmental conditions.

Chill availability was calculated in CP (Dynamic model) for a period between October 1st and February 18^th^. This is the first flowering date, registered in Zaragoza for the cultivar ‘Earliqueen’ in 2019. Temperatures at the end of February are warm during the day, reaching values above 20°C in both regions, thus plum cultivars are at flowering or about to bloom.

### 2.7 Probable impacts of climate change on fulfillment of chilling requirements

We assessed the possible impacts of climate change on the cultivation of the Japanese plum-type cultivars in the regions of Zaragoza and Badajoz by calculating the probability of fulfilling the chilling requirements of the 21 cultivars in future scenarios. We followed the methods described by Delgado et al. (2021) and Fadón et al. (2023) that account for uncertainty around the estimation of CR. In brief, we randomly sampled 1000 values within the range “mean CR ± sd”. We then computed the probability of CR fulfillment as the share of seasons (out of 100) for which the estimated chill availability exceeds each of the sampled chilling requirements. We summarized the results of this analysis by computing the median probability of CR fulfillment across 1000 values.

### 2.8 Data processing, analyses, and visualization of results

All statistical analyses were performed in the integrated development environment RStudio version 1.2.5033 (RStudio-Team, 2020) for the R programming language version 3.6.3 (R Core Team, 2021). The non-parametric Kruskal-Wallis test was chosen for multiple comparisons of chilling and heating requirements values that were not normally distributed.

Correlations between the variables CH, CU, CP, GDH, endodormancy release date (ER), and full bloom date (F50) were calculated using the Spearman correlation network in locations with data from multiple years. Corrr package (version 0.4.4) for R (Kuhn et al., 2020) was used for the correlation analysis and visualization.

For agro-climatic analyses, we used the chillR package (version 0.73.1) for R (Luedeling et al., 2023). For visualizing the results, we used the ggplot2 library (Wickham, 2011).

## 3 Results

### 3.1 Agroclimatic metrics in two regions in Spain

Badajoz, located in the southwestern region of Spain, experiences a Mediterranean climate characterized by hot, dry summers and mild, relatively wet winters. Winters are generally mild, with temperatures rarely dropping below freezing, although occasional cold snaps can occur. Zaragoza, located in northeastern Spain, experiences a semi-arid climate with hot summers and cool winters. inters are relatively mild, with temperatures usually staying above freezing, although occasional cold spells can occur, particularly in December and January. Summers in both locations are typically long and hot, with temperatures often exceeding 30°C, and occasional heatwaves can push temperatures even higher (AEMET, 2024). Slight differences occur among the different locations analyzed in Zaragoza: Montañana and La Almunia present very similar conditions while the climate in Caspe is slightly warmer due to a closer influence of the Mediterranean Sea and its low altitude.

**Figure 1:**
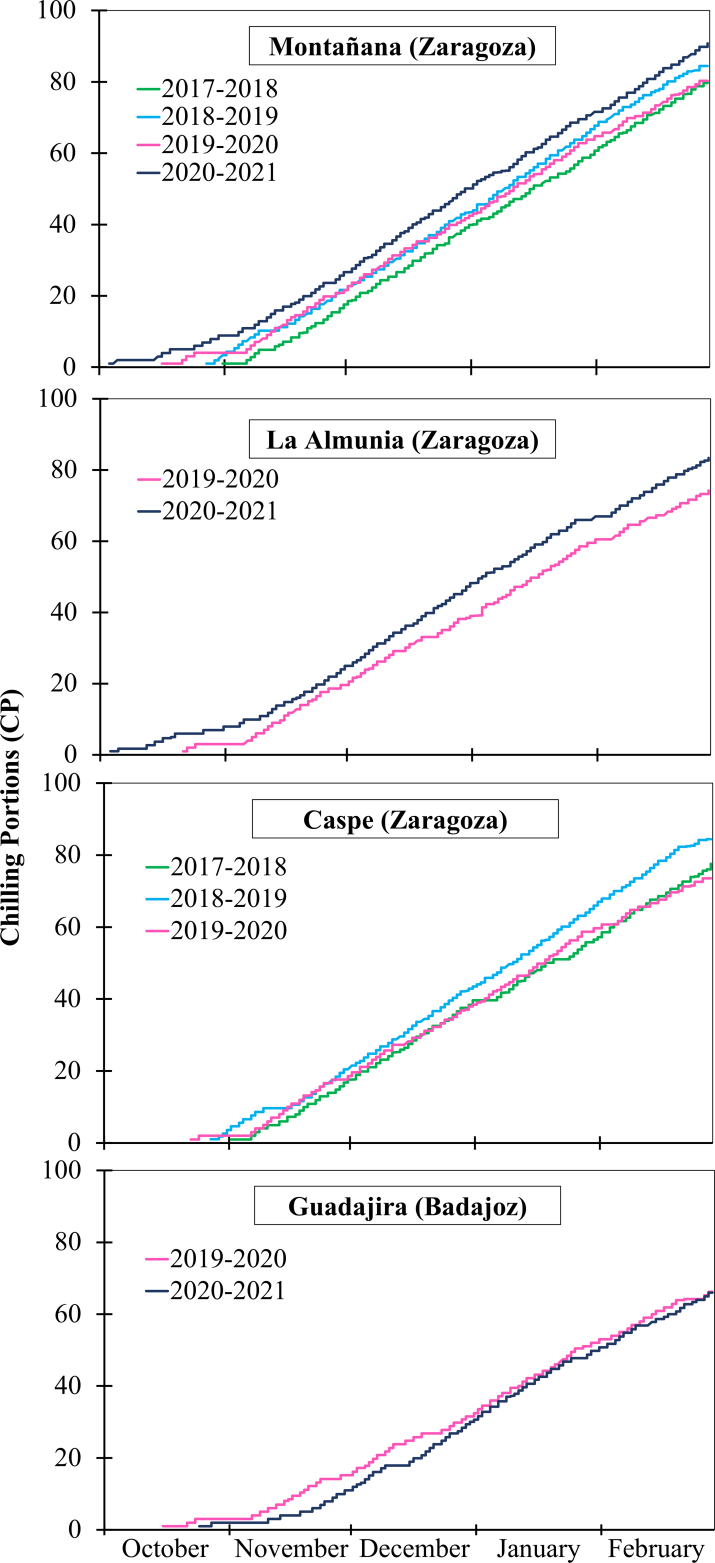
Chill accumulation according to the Dynamic model (chilling portions) in four locations over several winters.

Chill accumulation began in all locations and years between the second half of October and the first week of November. As an example, CP and CH began accumulating the second week of October in the four locations during the winter of 2019-2020, and CU from the first week of November. However, the amount of chill accumulated exhibited significant variations among regions, years, and models over the years of experiments (**Figure 1**). According to the three models, chill accumulated more quickly in Montañana, Zaragoza (1,101 CH, 1,285 CU, and 65 CP, January 31, 2020), while the mildest winter occurred in Guadajira, Badajoz (538 CH, 888 CU, 53 CP, January 31, 2020). In Caspe and La Almunia, chill accumulation followed a similar pattern in CH and CP, although more CU accumulated in Caspe than in La Almunia. According to the CP model, in Montañana and La Almunia, the coldest winter was 2020-2021, and the warmest was 2017-2018. In Caspe, the winter of 2018-2019 was colder than the other two winters (2017-2018, 2019-2020). In Guadajira, chill accumulation was similar in the two winters (2019-2020, 2020-2021), with 66 CP accumulated by January 31 (**Figure 1**).

### 3.2 Endodormancy release and chilling requirements

**Figure 2:**
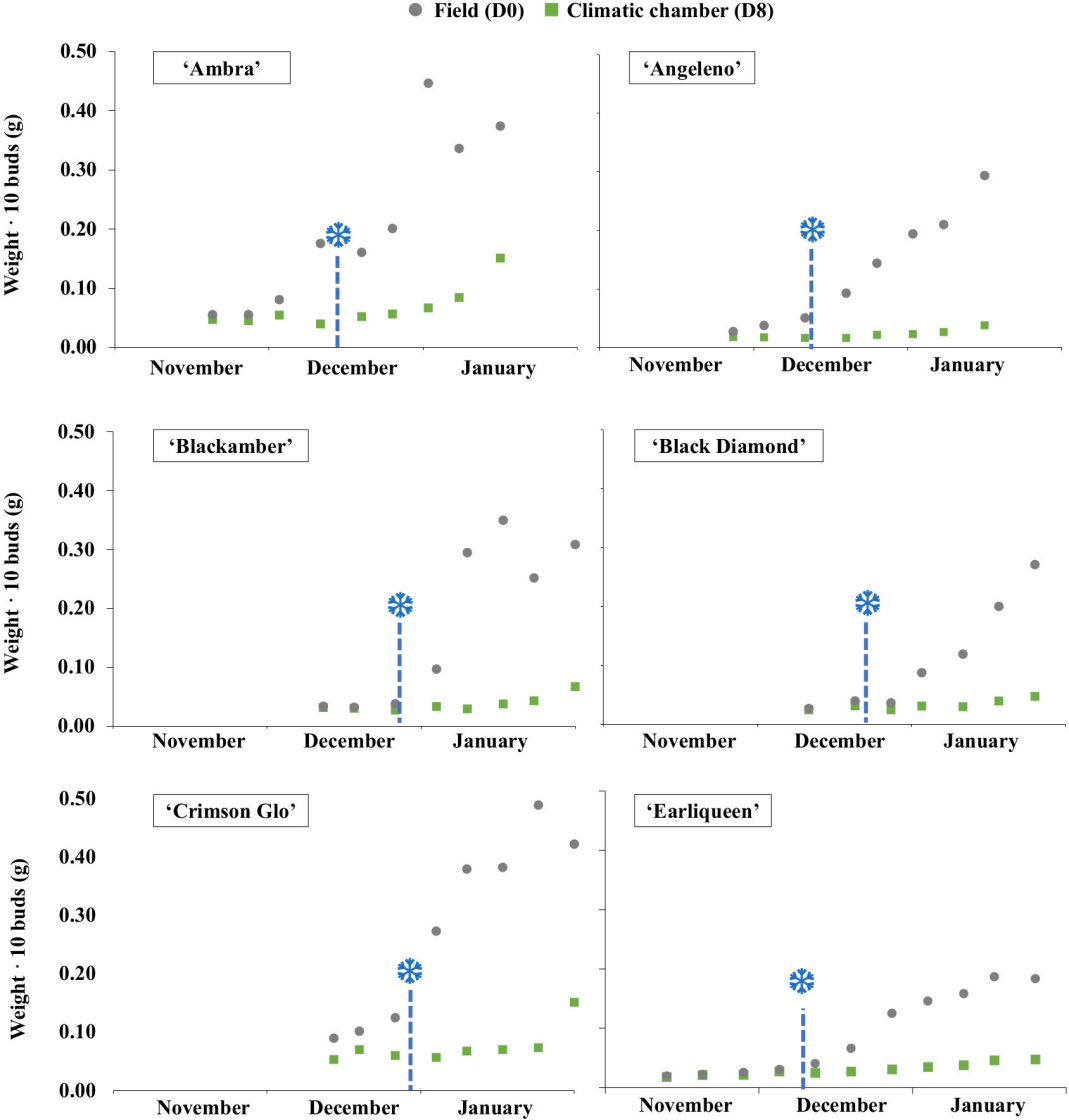
Growth of flower buds in six Japanese plum cultivars (‘Ambra’, ‘Angeleno’, ‘Blackamber’, ‘Black Diamond’, ‘Crimson Glo’ and ‘Earliqueen’) from November to January. Endodormancy release (❆) corresponds to a 30% increase in bud weight after 8 days in a forcing chamber with controlled temperature conditions.

The evaluation of bud weight and phenology in each cultivar allowed the characterization of their growth during dormancy phases under field conditions. At the beginning of winter, the average weight of 10 buds was approximately 0.02 g in all cultivars and remained relatively unchanged during November and mid-December. The weight increased to 0.25 g during budbreak (**Figure 2**, **Supplementary Figure S1**), corresponding to the phenological shift from stage A to B (Baggiolini, 1952).

The characterization of bud growth after a week in the forcing chamber allowed determining the end of the endodormancy period. The cultivar ‘Red Beaut’ was the earliest to release from endodormancy (December 2, 2020, Montañana, Zaragoza), while ‘Ambra’ was the latest (February 7, 2019, Guadajira, Badajoz). Endodormancy release occurred between December 4 and January 7 in La Almunia, between December 5 and January 24 in Caspe, between December 2 and January 3 in Zaragoza, and between December 30 and February 7 in Guadajira. Cultivars showed differences in endodormancy release between locations, ranging from 15 to 30 days, occurring earlier in Zaragoza (Caspe, La Almunia, and Montañana) than in Badajoz (Guadajira) (**Supplementary Table S1**).

After establishing the endodormancy period for each cultivar, year, and location, we determined the chilling requirements by quantifying the accumulated chill according to the three temperature models (**Supplementary Table S1**). The results showed a wide range of chilling requirements, 277-851 CH, 412-1,030 CU, and 26-51 CP, with significant differences (p<0.05) among cultivars. ‘Earliqueen’ (292 CH, 475 CU, 30 CP) had the lowest chilling requirements, while ‘Golden Globe’ (725 CH, 916 CU, 50 CP) showed the highest chilling requirements (**Supplementary Table S1**). Variability in chilling requirements was observed between years in the three models for each cultivar. The Weinberger model (CH) had higher coefficients of variation (CV, %) between years than the Utah (CU) and Dynamic (CP) models. No significant differences in chilling requirements were observed among locations and years for each cultivar.

Cultivars were classified into three groups based on their chilling requirements. The only ‘low chilling requirements’ cultivar was ‘Earliqueen’. Thirteen cultivars were grouped as ‘medium chilling requirements’ cultivars, and the other seven as ‘high chilling requirements’ cultivars (**Table 1**).

**Table 1:** Classification of 21 Japanese plum cultivars based on their chilling requirements in chill portions (CP), chill hours (CH), and chill units (CU).

Chilling requirements	Range	Cultivars
Low	≤ 30 CP	Earliqueen
	≤ 300 CH	
	≤ 500 CU	
Medium	31-40 CP	Ambra, Angeleno, Blackamber Black Diamond, Crimson Glo, Fortune, Freedom, John W., Laetitia, Red Beaut, Santa Rosa, Songold, 606
	301-500 CH	
	501-700 CU	
High	≥ 41 CP	Golden Globe, Golden Japan, Golden Plumza, Hiromi Red, Joanna Red, Owen T., TC Sun
	≥ 501 CH	
	≥ 701 CU	

### 3.3 Ecodormancy and heating requirements

The ecodormancy periods ranged from the end of endodormancy to full flowering for each cultivar, year, and location. The full flowering dates (F50) showed variability among cultivars and years, ranging from February 18, 2019, for the earliest cultivar (‘Earliqueen’) to March 16, 2019, for the latest (‘Golden Plumza’). Flowering duration was shorter in La Almunia (from February 18 to February 25), Caspe (from February 19 to March 11), and Montañana (from February 24 to March 14) compared to Guadajira, Badajoz (from February 23 to March 16). However, no significant differences were observed between locations for cultivars analyzed in two locations (**Supplementary Table S1**).

After establishing the ecodormancy period for each cultivar, year, and location, we determined the heating requirements by quantifying the accumulated heat from endodormancy release to full bloom according to the Growing Degree Hours (GDH) model. Heating requirements ranged from 4,343 (‘Ambra’) to 9,525 GDH (‘John W.’) (**Supplementary Table S2**). Variability in heating requirements was observed among years and locations in all cultivars, which were classified into three groups based on their heating requirements. The cultivars ‘606’, ‘Ambra’, ‘Black Diamond’, ‘Joanna Red’ and ‘Owen T’ were classified as ‘low heating requirements’ cultivars. ‘Blackamber’, ‘Freedom’, ‘Golden Plumza’, ‘Laetitia’, ‘Red Beaut’, ‘Santa Rosa’, ‘Songold’ and ‘TC Sun’ were classified as ‘medium heating requirements’, and the other eight cultivars as ‘high heating requirements’ (**Table 2**).

**Table 2:** Classification of 21 Japanese plum cultivars based on their heating requirements in growing degree hours (GDH).

Heating requirements	Range	Cultivars
Low	≤ 6,500 GDH	606, Ambra, Black Diamond, Joanna Red, Owen T
Medium	6,501-7,000 GDH	Blackamber, Freedom, Golden Plumza, Laetitia, Red Beaut, Santa Rosa, Songold, TC Sun
High	> 7,000 GDH	Angeleno, Crimson Glo, Earliqueen, Fortune, Golden Globe, Golden Japan, Hiromi Red, John W

### 3.4 Correlations between variables

**Figure 3:**
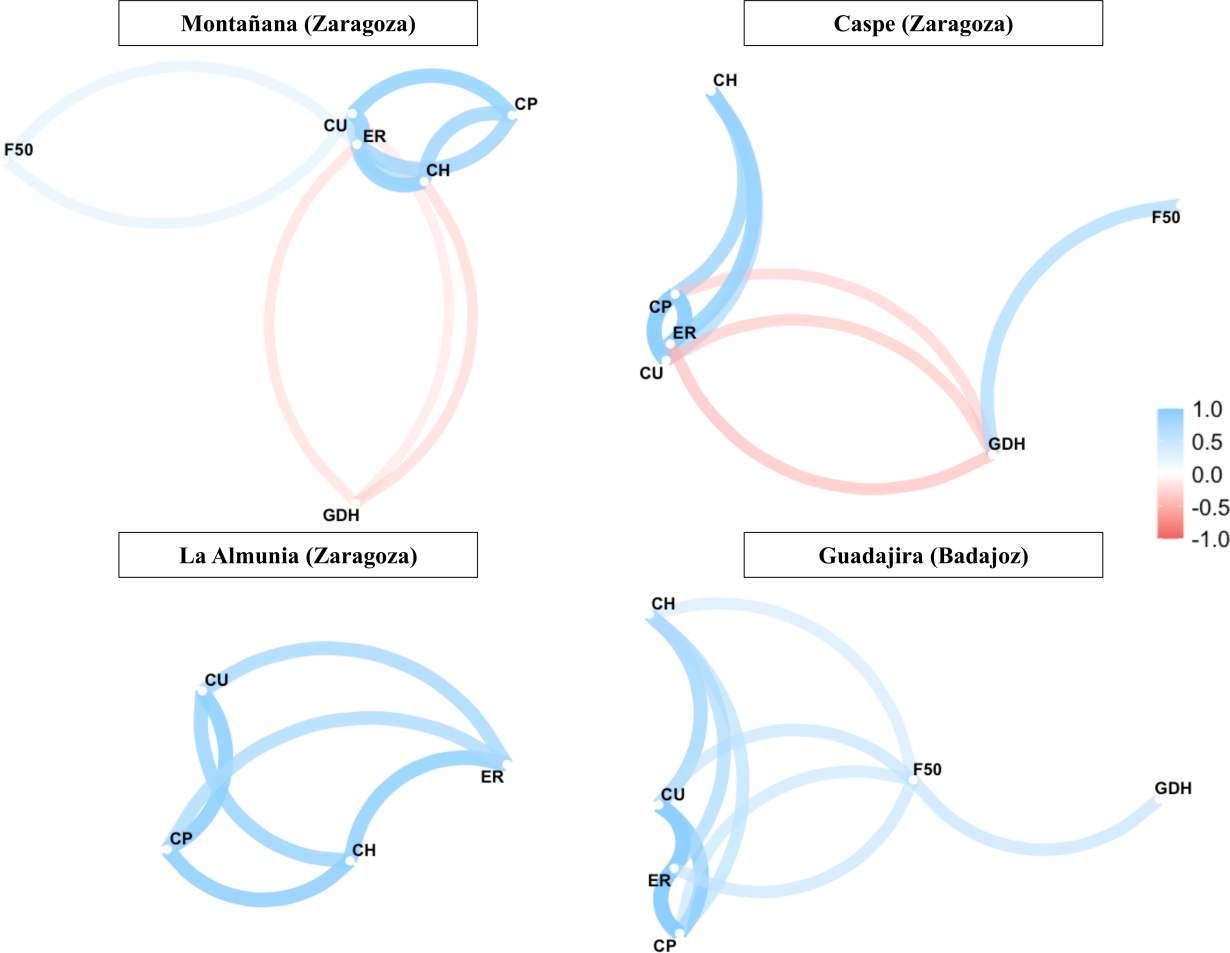
Spearman correlation network between chilling hours (CH), chilling units (CU) and chilling portions (CP), growing degrees hours (GDH), endodormancy release date (ER) and dates of full bloom (F50) of 21 accessions of Japanese plum-type cultivars analyzed in four locations.

In all four locations, endodormancy release dates were highly and positively correlated with chilling requirements estimated in the three models. Chilling requirements according to the three models were positively correlated with each other. In Caspe and Montañana, heating requirements were negatively correlated with chilling requirements, and these correlations were not observed in Guadajira. The full flowering date (F50) was correlated with different variables in each location: in Caspe, positively with heating requirements; in Montañana, positively with chilling requirements (in different intensity depending on the model); and in Guadajira, it was significantly correlated with chilling and heating requirements (**Figure 3**).

### 3.5 Historic and future winter chill in Badajoz and Zaragoza regions

**Figure 4:**
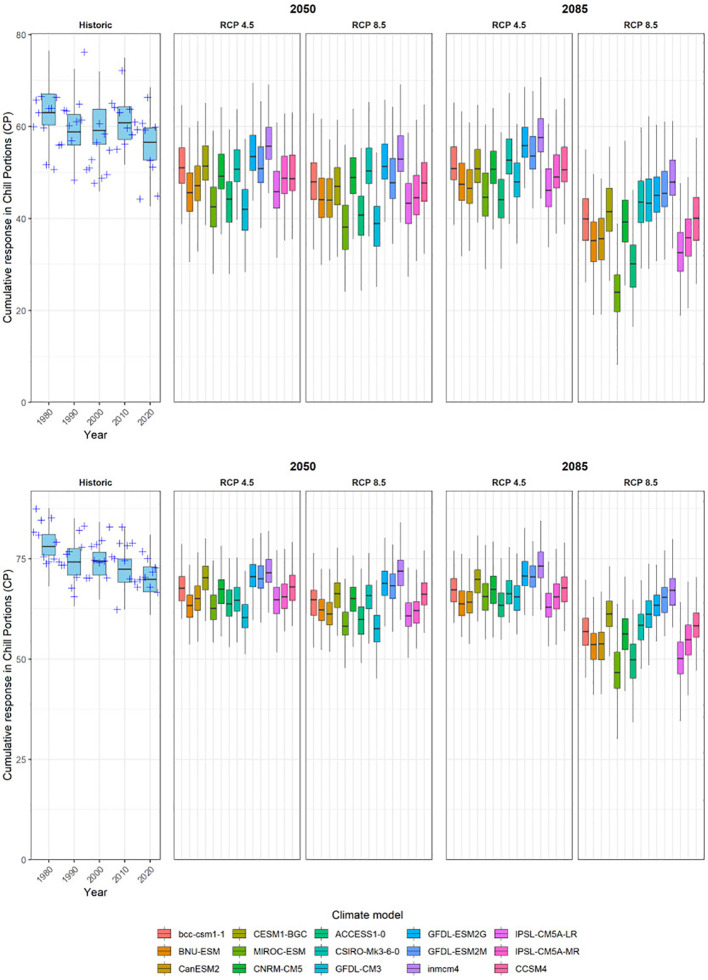
Historic and projected future winter chill accumulated till the first flowering date (February 13^th^) in Guadajira (Badajoz, Spain) (above) and Zaragoza, Spain (below). The “Historic” panel shows the actual chill accumulation between 1974 and 2022 (crosses) and historic simulated weather scenarios for the reference years 1980, 1990, 2000, 2010, and 2020 (boxplots). The remaining four panels show chill accumulation under future scenarios defined by two Representative Concentration Pathways (RCP4.5 or RCP8.5) by two-time horizons (2050 and 2085). In each of these panels, boxplots illustrate the plausible chill distributions produced by a weather generator according to temperature projections by 15 climate models.

Chill accumulation considerably decreased from the 80´s of 20^th^ century to nowadays in both regions, Badajoz and Zaragoza (**Figure 4**). Chill accumulation on the simulated historic scenarios for the reference year 1980 ranged between 50.7 and 57.1 CP (25th - 75th interval) in Badajoz, and between 61.2 CP and 67 CP in Zaragoza. For 2020, chill accumulation of the simulated scenarios ranged between 44.6 CP and 50.7 CP in Badajoz, and 53.8 - 59 CP in Zaragoza.

The trend of decreasing chill accumulation is expected to persist in all scenarios and models. In Badajoz, the predictions for 2050 did not vary between the RCP 4.5 and RCP 8.5 scenarios, but the differences between the two scenarios became significant by 2085. While under RCP 4.5 the chill accumulation in 2085 remained similar or slightly higher in 2050, under RCP 8.5 chill accumulation was predicted to decrease drastically in all models by 2085, particularly in the MIROC-ESM model. Chill was also predicted to reduce in Zaragoza, but the decrease was not as accentuated as in Badajoz. Similarly, chill accumulation in 2050 was similar under both scenarios, and in 2085 under RCP 4.5 chill accumulation did not vary or even increased, but under RCP 8.5 the reduction of chill was very accentuated.

### 3.6 Chilling fulfillment of cultivars in past and future climates

**Figure 5:**
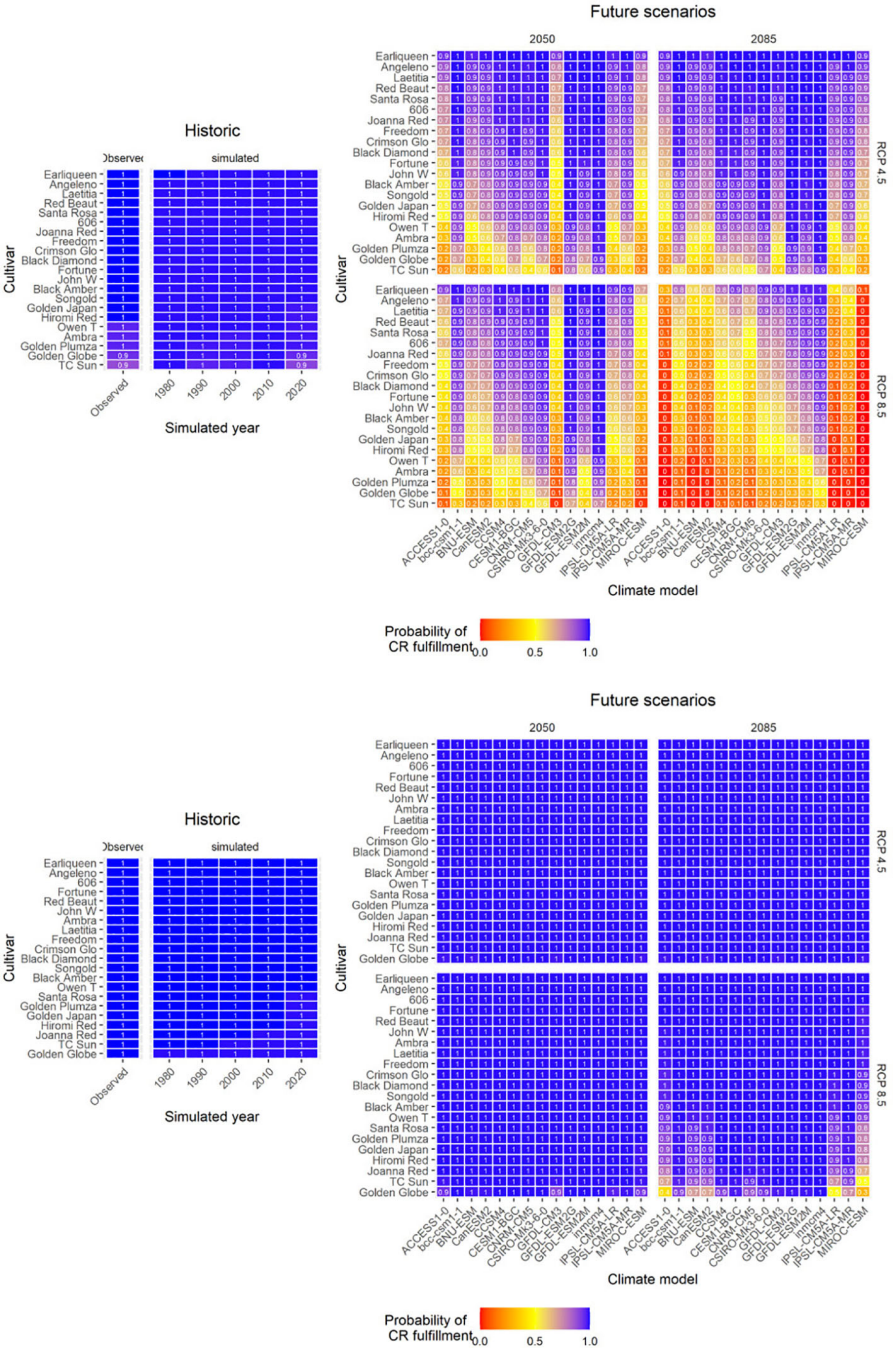
Probability of satisfying the estimated cultivar-specific chilling requirements (calculated as Chilling Portions) of 21 cultivars of Japanese plums in Guadajira (Badajoz, Spain) (above) and Zaragoza, Spain (below) for two RCP scenarios (RCP4.5 and RCP8.5), two time horizons (2050 and 2085) and 15 global climate models.

To predict the viability of these cultivars in the future, we determined the frequency of CR fulfillment as the likelihood of meeting these requirements in the future using the estimates of the cultivar-specific CR. In Zaragoza, our estimations indicated that these cultivars will be optimum to cultivate in the future, since chill accumulation will be fulfilled in all cases (**Figure 5**). However, in Badajoz, important attention should be given to the selection of cultivars in the design of new orchards in the future. Most global climate models predicted a relatively low-risk profile (80%-100%) for cultivars with low to medium CR under the RCP 4.5 scenario. While cultivars such as ‘Golden Plumza’, ‘Golden Globe’, and ‘TC Sun’ have a high risk of CR unfulfillment (10-40%) under the RCP 4.5 scenario. The RCP 8.5 scenario presented a more dramatic situation. By the end of this century about 15 out of 21 cultivars would present a high risk of CR unfulfillment, and only the cultivar ‘Earliqueen’ presented a low-risk profile (**Figure 5**).

## 4 Discussion

The agroclimatic requirements of 21 Japanese plum-type cultivars, including 12 that have been analyzed for the first time, were examined in this study. Previous data available for Japanese plum were limited to 11 cultivars analyzed in Murcia (Ruiz et al., 2018) and seven in Zaragoza (Tabuenca, 1967), with nine of them being included in this study. Six cultivars showed chilling requirements similar to those obtained in previous works (‘Black Diamond’, ‘Fortune’, ‘Golden Globe’, ‘Laetitia’, ‘Red Beaut’, and ‘Santa Rosa’). In ‘Angeleno’ and ‘Songold’, significantly different values (>20%) were obtained compared to previous results (Ruiz et al., 2018), while in ‘Golden Japan’, the results were similar to the previous work carried out in Murcia (Ruiz et al., 2018) but different from the other study analyzed in Zaragoza (Tabuenca, 1967). Differences in chilling requirements among locations have been observed in numerous previous studies on various stone fruit species [reviewed in Fadón et al. (2020b)], which can be attributed to methodological limitations (Luedeling et al., 2009) and differences in experimental methodology, such as sampling frequency, growth chamber conditions, and the parameters considered for bud growth assessment, making it challenging to compare results across different studies (Dennis, 2003). However, in such cases, the data can be valid for comparing chilling requirements among cultivars and for assessing the adaptation of cultivars to the study area (Fadón and Rodrigo, 2018).

In contrast to the other two chilling quantification models (CH and CU), the dynamic model (CP) showed a lower mean CV between years and locations for each cultivar. Therefore, this model appears to be the most suitable for the conditions of these two regions in Spain. Previous studies have shown that the dynamic model is the most appropriate for warm or temperate winter regions (Campoy et al., 2011; Fadón et al., 2020b).

Distinct characteristics of Japanese plum have influenced the experimental design previously used in other species. Firstly, the bud size is up to 5-10 times smaller than in other *Prunus* species (Guerra and Rodrigo, 2015), making it necessary to monitor a larger number of buds. Secondly, in most cultivars, it was necessary to sample longer shoots (>30 cm), as in previous work on apricot (Campoy et al., 2012) and Japanese plum (Ruiz et al., 2018), than those used in other studies on Japanese apricot (Gao et al., 2012), sweet cherry (Fadón et al., 2018b), and peach (Citadin et al., 2001). Furthermore, phenological development after the period in the forcing chamber was highly heterogeneous among cultivars. In most cases, the first phenological change observed in the flowers buds occurred once a weight increase of more than 30% had been achieved. Therefore, evaluating phenology as an indicator of chilling requirement fulfillment can result in overestimating chilling requirements in some cultivars. Bud weight assessment appears to be a more reliable indicator than phenological development in most Japanese plum cultivars.

Chilling requirements were lower than those of other *Prunus* species such as apricot (29.8-78.9 CP) (Ruíz et al., 2007; Campoy et al., 2012), sweet cherry (29-107 CP) (Alburquerque et al., 2008; Fadón et al., 2021b), European plum (579-1323 CH) (Tabuenca, 1967), and peach (1-122 CP) (Citadin et al., 2001), but higher than those of almond (3.4-55.4 CP) (Egea et al., 2003; Alonso et al., 2005). The variability observed among the analyzed cultivars is similar to that found in other *Prunus* species, with the exception of peach, which exhibits a wider range (Fadón et al., 2020b).

The accumulated chill in all locations proved to be sufficient for endodormancy release in all evaluated cultivars so far. However, special attention should be paid to the cultivation of Japanese plum in Badajoz, which nowadays produces about 48% of Spanish plums (>78,500 t in 5,000 ha) (MAPA, 2023). Our predictions revealed failures in CR fulfilment in most cultivars, which would not successfully adapt to future climatic conditions in this region. In Zaragoza, which produced the 7% of Japanese plums in Spain (>11,000 t in 1,400 ha) (MAPA, 2023), future predictions revealed successful fulfilment of the CR at least until the end of the 21st century.

The flowering dates of the cultivars in this study were similar to those in Murcia (Ruiz et al., 2018) and earlier than in Serbia (Ruml et al., 2021). In general, Japanese plum flowering occurs earlier than sweet cherry (Alburquerque et al., 2008; Fadón et al., 2021b) but later than apricot (Campoy et al., 2012) and almond (Alonso et al., 2005). Heating requirements were similar to those determined in a previous Japanese plum study (Ruiz et al., 2018), but with a wider range, likely due to the larger number of cultivars analyzed in this study. They also exhibited a wider range than in apricot (Ruíz et al., 2007; Campoy et al., 2012), sweet cherry (Alburquerque et al., 2008; Fadón et al., 2021b), and almond (Egea et al., 2003; Alonso et al., 2005). As expected, chilling requirements according to all three models were related to the endodormancy release date. Heating requirements negatively correlated with chilling requirements, showing that cultivars with lower chilling requirements needed more heat accumulation to flower properly. The full bloom date was more influenced by heating requirements in the colder conditions of Caspe (Zaragoza), similar to almond (Alonso et al., 2005) and sweet cherry (Fadón et al., 2021b) grown under similar conditions. In the warmer conditions of Guadajira (Badajoz), flowering was more influenced, closely and positively, by chilling requirements, as in the climatic conditions of southeastern Spain for apricot (Campoy et al., 2012), almond (Egea et al., 2003), and Japanese plum (Ruiz et al., 2018).

The determination of the agroclimatic requirements of a wide and diverse group of cultivars from different breeding programs, allowed the assessment of their potential adaptation to each cultivation area. However, the differences observed between the studied areas and results from previous studies emphasize the need for standardization of experimental conditions to accurately determine the endodormancy release time. In other species, different biological (Julián et al., 2011, 2014; Fadón et al., 2018a, 2019, 2021a) and molecular markers (Bielenberg et al., 2008; Yamane et al., 2011; Balogh et al., 2019; Vimont et al., 2019; Quesada-Traver et al., 2020) have been proposed to determine the transition from endodormancy to ecodormancy. Identifying a biomarker for endodormancy release in Japanese plum would allow for the validation of the results obtained and facilitate the estimation of agroclimatic requirements in the future.

Winter chill accumulation is expected to decrease both in Badajoz and Zaragoza, as reported in many other regions around the world (Luedeling et al., 2009; Fernandez et al., 2020; Delgado et al., 2021; Fadón et al., 2023), with an accentuated warmer trend in Mediterranean areas as Spain (Fernandez et al., 2022; Fadón et al., 2023). In Zaragoza, the predicted winter temperatures seem to be sufficient for all the analyzed cultivars to fulfill their chilling requirements in the two global warming scenarios (RCP4.5 and RCP8.5) and in the two time horizons. However, the situation may be worse in Badajoz, where it is foreseeable that all the cultivars studied will have problems fulfilling their chilling requirements in many years in both warming scenarios. Thus, in Badajoz only those cultivars with low chilling requirements could be grown, while in Zaragoza, some of the high chilling cultivars could have production problems. This highlights the importance of knowing the agroclimatic requirements of new cultivars, as well as of those already established, to anticipate production problems in warmer winter years. The cultivation of the most high chilling cultivars could shift to colder areas but with a higher risk of frost during flowering (Luedeling, 2012). On the other hand, the new cultivars with the greatest potential to adapt to the new conditions caused by global warming in most current growing areas would be those with low chilling requirements, which is a common objective in several breeding programs (Topp et al., 2008; Ruiz et al., 2019). It is foreseeable that the cultivation of low chilling cultivars will become increasingly important in the coming decades, both in traditional regions and in the new warmer areas due to the expansion of Japanese plum cultivation, as is happening in other fruit crops such as apple (Delgado et al., 2021) or sweet cherry (Fadón et al., 2023). Although the impact on the agronomic behavior of these cultivars in these situations cannot be determined exactly, it is probably that fruit production may be compromised, which would have a great economic impact, since Badajoz is the main plum producing region in Spain and one of the main ones worldwide (Guerra et al., 2020). When a cultivar has not fulfilled its chilling requirements at the time it normally blooms, the trees theoretically could continue to accumulate chill (Fadón et al., 2018b). However, even if the chilling requirements were eventually fulfilled, this would have a marked impact on the flowering date, potentially causing a lack of overlap between pollinating and pollinated cultivars, with the consequent lack of fruit set and production, since most Japanese plum cultivars are self-incompatible and require cross-pollination to fertilize their flowers (Guerra and Rodrigo, 2015; Guerra et al., 2020). On the other hand, if cultivars fail to fulfill their chilling requirements, irregular and heterogenous bud burst and bloom can occur, leading to lack fruit set and fruit production (Erez, 2000; Fadón et al., 2020a, 2021b; Fernandez et al., 2022).

## Data availability statement

The raw data supporting the conclusions of this article will be made available by the authors, without undue reservation.

## Author contributions

BG: Conceptualization, Data curation, Formal analysis, Methodology, Visualization, Writing – original draft, Writing – review & editing. EF: Conceptualization, Data curation, Formal analysis, Investigation, Methodology, Visualization, Writing – original draft, Writing – review & editing. MG: Conceptualization, Data curation, Funding acquisition, Resources, Validation, Visualization, Writing – original draft, Writing – review & editing. JR: Conceptualization, Formal Analysis, Funding acquisition, Investigation, Project administration, Resources, Supervision, Validation, Visualization, Writing – original draft, Writing – review & editing.

## Supplementary material

Supplementary Figure 1 Growth of flower buds in fifteen Japanese plum cultivars (‘Fortune’, ‘Freedom’, ‘Golden Globe’, ‘Golden Japan’, ‘Golden Plumza’, ‘Hiromi Red’, ‘Joanna Red’, ‘John W’, ‘Laetitia’, ‘Owen T’, ‘Red Beaut’, ‘Santa Rosa’, ‘Songold’, ‘TC Sun’, ‘606’) from November to January. Endodormancy release (❆) corresponds to a 30% increase in bud weight after 8 days in a forcing chamber with controlled temperature conditions.

Supplementary Table 1 Japanese plum cultivars assayed including the pedigree, origin, main agronomic traits (ripening time, skin color and flesh color), chilling requirements analyzed in Guadajira (Badajoz) and Caspe, La Almunia, and Montañana (Zaragoza) expressed in chilling hours (CH), chilling units (CU), and chilling portions (CP), and heating requirements expressed in growing degree hours (GDH). Values obtained for each year, the mean, and the coefficient of variation (CV) (%) between years and locations (in italics) are shown. ns: non significant at p < 0.05. OP: Open pollination.
