# Supplementary material for: Perspectives on the adaptation of Japanese plum-type cultivars to reduced winter chilling in two regions of Spain
Source: Front Plant Sci. 2024 Apr 17;15:1343593. doi: 10.3389/fpls.2024.1343593 (PMC11061358; doi:10.3389/fpls.2024.1343593)
Supplement: Supplementary Figure 1 — Growth of flower buds in fifteen Japanese plum cultivars (‘Fortune’, ‘Freedom’, ‘Golden Globe’, ‘Golden Japan’, ‘Golden Plumza’, ‘Hiromi Red’, ‘Joanna Red’, ‘John W’, ‘Laetitia’, ‘Owen T’, ‘Red Beaut’, ‘Santa Rosa’, ‘Songold’, ‘TC Sun’, ‘606’) from November to January. Endodormancy release (❆) corresponds to a 30% increase in bud weight after 8 days in a forcing chamber with controlled temperature conditions. [file Image_1.pdf]

Supplementary Material

Perspectives on the Adaptation of Japanese Plum-type Cultivars to Reduced Winter Chilling in Two Regions of Spain

Brenda I. Guerrero*, Érica Fadón, M. Engracia Guerra, Javier Rodrigo

*** Correspondence:** Brenda I. Guerrero: brguerrero@uach.mx

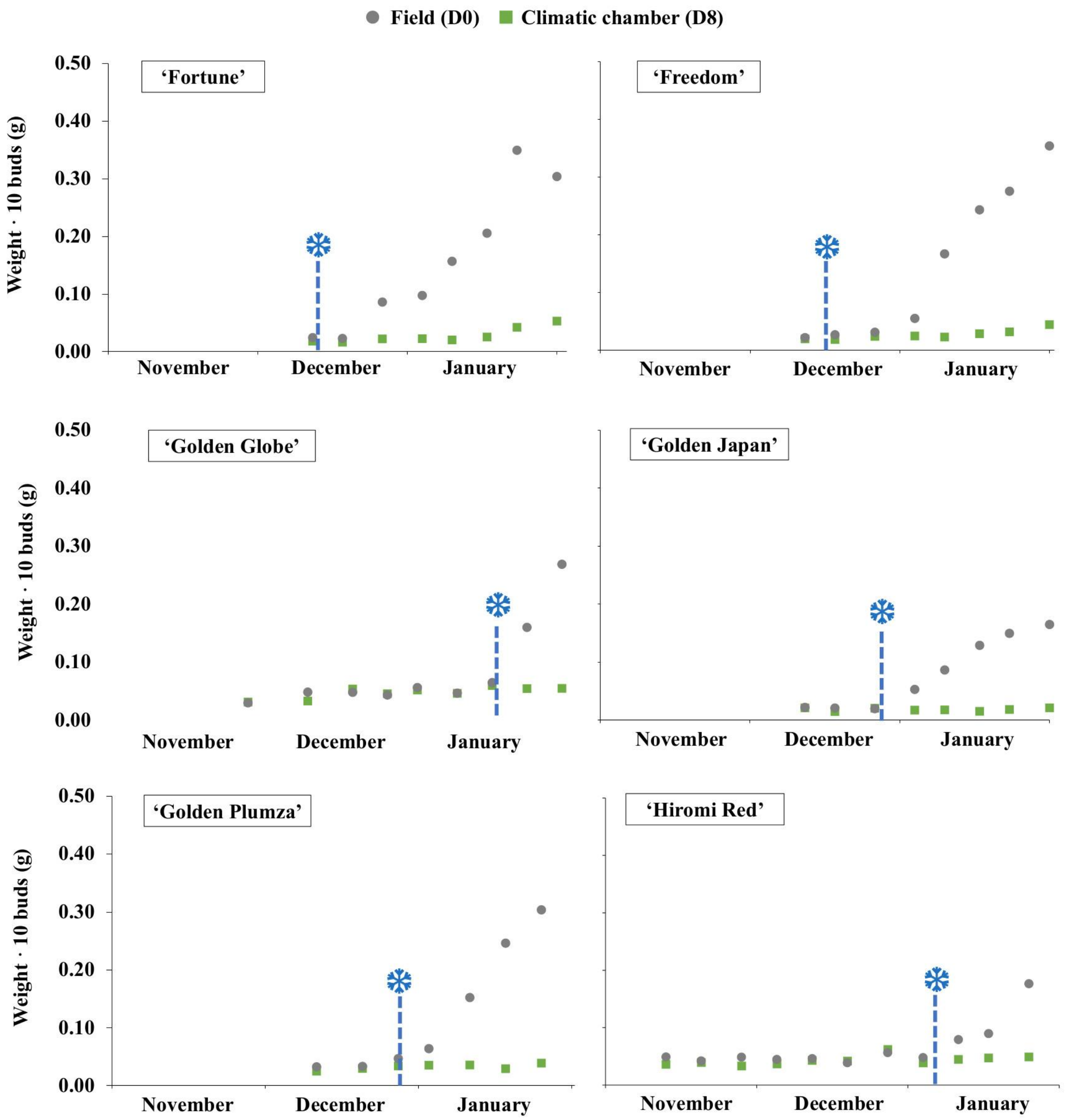
Field (D0)
Climatic chamber (D8)
'Fortune'
'Freedom'
'Golden Globe'
'Golden Japan'
'Golden Plumza'
'Hiromi Red'
Weight · 10 buds (g)
0.50
0.40
0.30
0.20
0.10
0.00
November
December
January

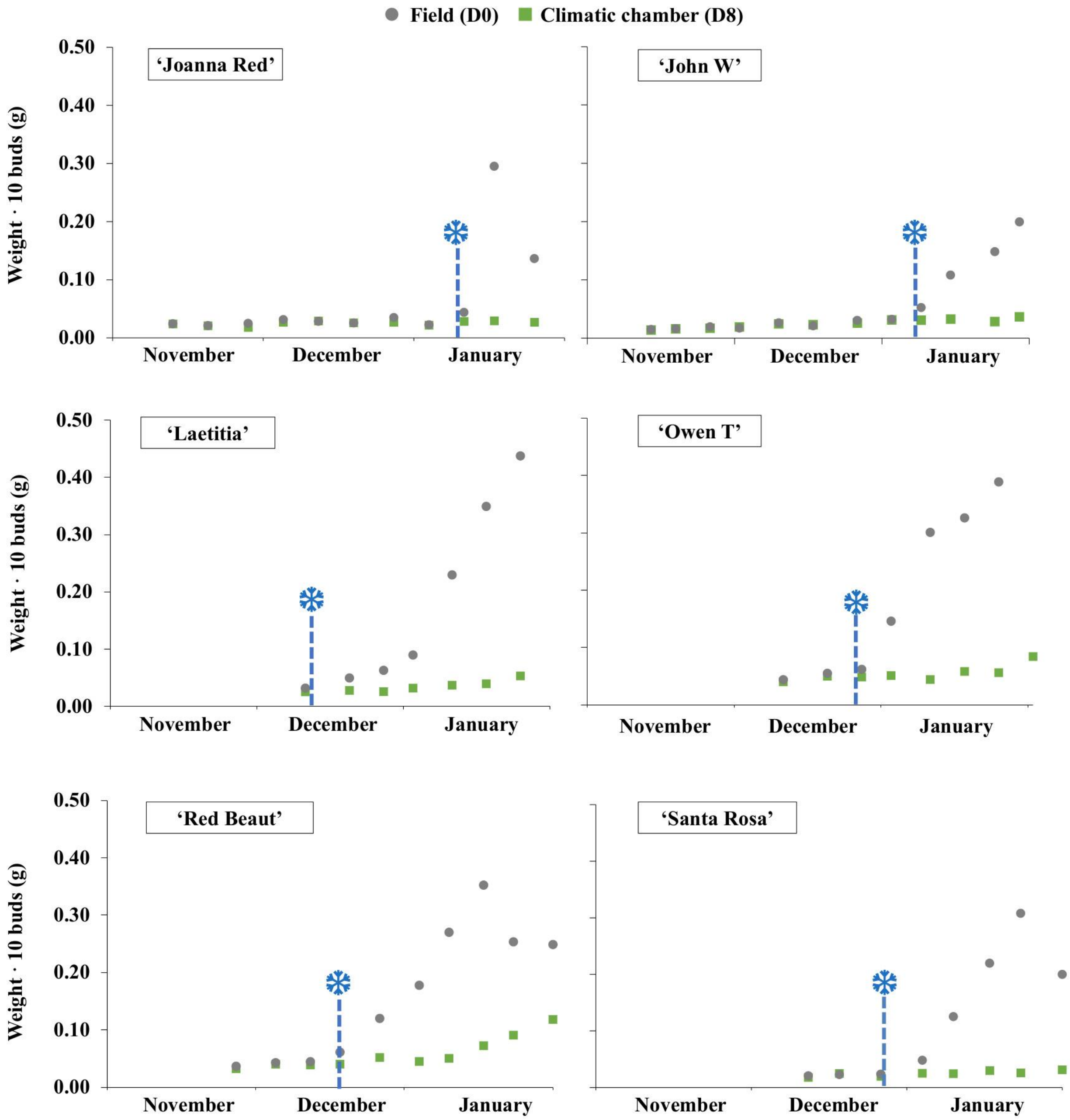
Field (D0)
Climatic chamber (D8)
'Joanna Red'
'John W'
'Laetitia'
'Owen T'
'Red Beaut'
'Santa Rosa'
Weight · 10 buds (g)
0.50
0.40
0.30
0.20
0.10
0.00
November
December
January

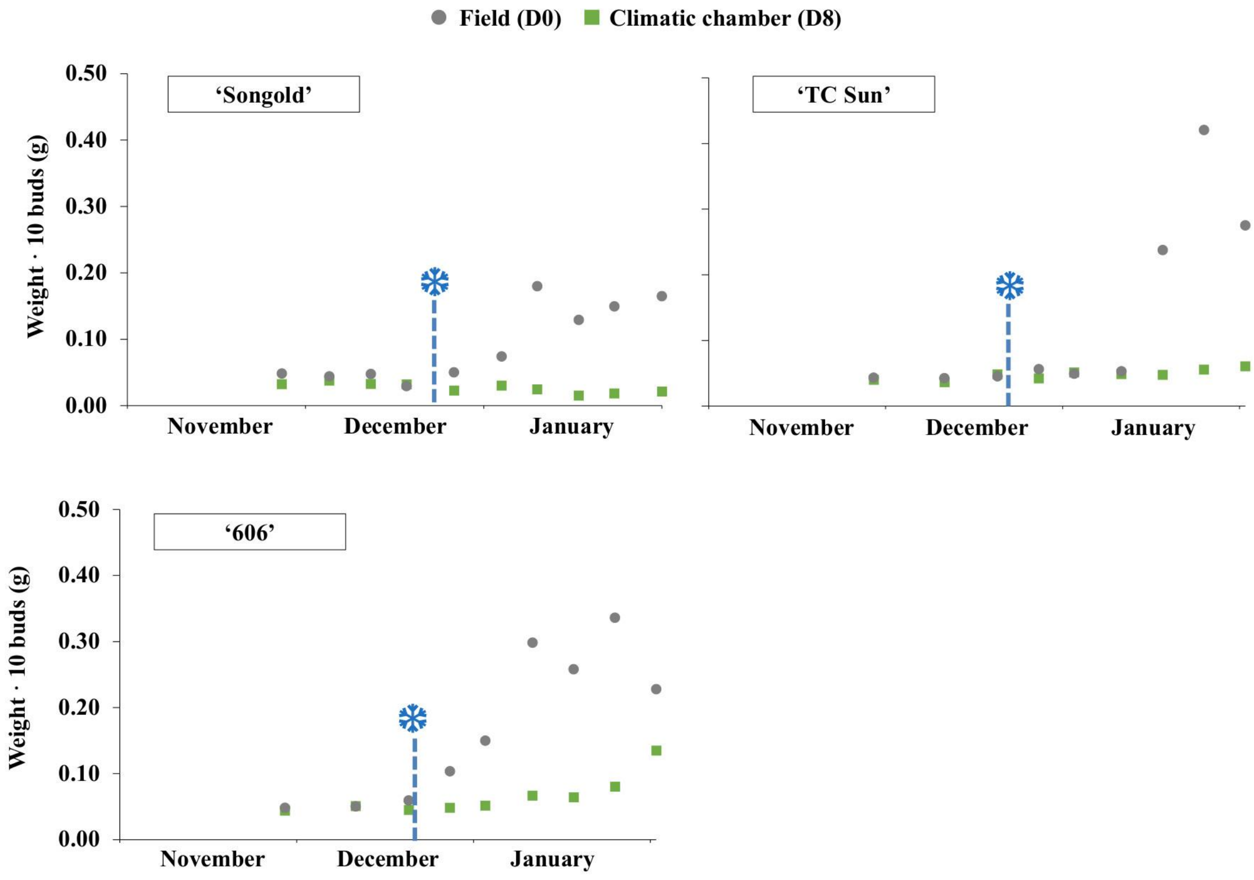

Figure S1. Growth of flower buds in fifteen Japanese plum cultivars ('Fortune', 'Freedom', 'Golden Globe', 'Golden Japan', 'Golden Plumza', 'Hiromi Red', 'Joanna Red', 'John W', 'Laetitia', 'Owen T', 'Red Beaut', 'Santa Rosa', 'Songold', 'TC Sun', '606') from November to January. Endodormancy release (❄) corresponds to a 30% increase in bud weight after 8 days in a forcing chamber with controlled temperature conditions.
